# Implementation of a Minimal Sedation Protocol for Patients With Developmental Disabilities and Needle Phobia

**DOI:** 10.7759/cureus.42154

**Published:** 2023-07-19

**Authors:** Julianna Rava, Kashia A Rosenau, Kendal Wilkie, Eric Curcio, Alice Kuo

**Affiliations:** 1 Medicine-Pediatrics, University of California Los Angeles David Geffen School of Medicine, Los Angeles, USA; 2 Medicine, University of California Los Angeles David Geffen School of Medicine, Los Angeles, USA

**Keywords:** needle anxiety, needle phobia, developmental disabilities, autism, minimal sedation

## Abstract

Objective

Patients with intellectual and developmental disabilities (IDD) experience needle phobia at greater rates than individuals in the general population. Needle phobia deters patients with IDD from receiving routine medical procedures, which impacts their physical health outcomes. The aim of this quality improvement study was to assess the feasibility of a minimal sedation protocol in an outpatient care setting for patients with IDD and needle phobia.

Methods

The sample included 18 patients characterized as having a diagnosis of IDD only or IDD and needle phobia compared to patients with only a diagnosis of needle phobia. Reasons for referral to intervention included routine lab work, therapeutic drug monitoring, and routine vaccination. The minimal sedation intervention involved intranasal administration of a benzodiazepine (midazolam) by a registered nurse. Outcomes of interest were administration of the sedation and administration of medical orders.

Results

Nearly a third of patients were children (33.3%, n=6), and 39% of patients were female (n=7). Individuals with IDD (including those both with and without needle phobias) comprised 72.2% of patients (n=13). Half of intervention encounters were successful in both administering the sedation and performing the medical orders (n=9). Among individuals with IDD, 38.4% successfully completed the intervention (n=5).

Conclusion

This pilot study assessed the feasibility of implementing a minimal sedation protocol in primary care outpatient care settings. The preliminary results suggest that the minimal sedation protocol may improve the uptake of needle-related medical procedures for patients with IDD and/or needle phobia. The minimal sedation protocol should be studied in a larger sample and among multiple outpatient settings to establish effectiveness of the intervention.

## Introduction

Nearly twice as many children and adults with intellectual and developmental disabilities (IDD) experience needle-related anxiety when visiting their primary care provider for routine care visits [1-3]. Furthermore, the rates of needle phobia for individuals with IDD exceed rates in the general population [1]. Needle phobia (also known as trypanophobia or blood-injection-injury phobia) is the intense fear of injections, transfusions, blood, or other medical care related to needles or injury [4]; it goes beyond general needle-related anxiety and promotes avoidant behavior toward the use of needles for medical procedures. Studies suggest that 15% of autistic children have a needle phobia compared to 5% of neurotypical children with needle phobia [2,3,5]. For individuals with IDD, needle phobia poses a significant risk to their physical health outcomes. Specifically, individuals with IDD that experience a needle phobia are less likely to maintain up-to-date vaccinations, such as for influenza or more recently COVID-19 [6,7]. For individuals with IDD that have diabetes or other chronic health conditions, a needle phobia would inhibit their ability to get routine blood draws and injections [8,9]. The inability to receive appropriate medical care can have dire outcomes for individuals with IDD, as more than a third of deaths for individuals with IDD are likely preventable with healthcare interventions [10]. Therefore, it is imperative to address needle phobia for individuals with IDD and provide a solution within primary care.

Current intervention techniques for needle phobia in healthcare include the use of behavioral interventions, distraction techniques, and topical anesthetics [11-19]. While these strategies enable medical procedures to be performed for many patients with needle-related anxiety, there is still a subpopulation of individuals with IDD whose severe needle phobia inhibits providers from performing routine care. Innovative strategies are necessary to enhance standard practices and increase the likelihood that this subset of individuals with IDD can tolerate important needle-related medical procedures, such as blood draws and vaccinations.

Sedation techniques have been used in dental care settings to care for patients with needle phobia. The American Society of Anesthesiologists Committee and the American Dental Association (ADA) established guidelines directly related to mild to moderate sedation in outpatient settings [20,21]. Specifically, minimal sedation techniques (also known as anxiolysis or procedural sedation) have been shown as an effective strategy to perform dental procedures for pediatric patients [22-24]. The ADA guidelines recommend the use of low-dose and fast-acting benzodiazepines when performing minimal sedation, which have a high therapeutic index and low dose-response curve [21,25]. Specifically, midazolam (brand name Versed) has been recognized as a successful minimal sedation treatment since the agent has anxiolytic and amnestic properties and the maximal effect is reached within 15-20 minutes but can last up to two hours [14]. While minimal sedation has been a norm in dental offices, it is only recently implemented to help individuals with IDD and/or individuals with needle phobia complete routine care procedures [14]. However, current minimal sedation protocols typically involve day visits to pediatric hospitals, thereby requiring a substantial amount of skill, time, cost, and resources. To our knowledge, there have not been any protocols developed to enable minimal sedation procedures for needle phobia in the outpatient, primary care setting.

Study aims

The aim of this pilot study is to assess the feasibility of a novel minimal sedation protocol that provides needle-related medical care to patients in their preferred outpatient care setting. The minimal sedation protocol is a part of a larger initiative, the Needle Anxiety Program, that aims to assist patients with IDD with needle-related anxiety in primary care settings. This quality improvement report states the one-year preliminary outcomes since implementing the program and describes the development of the minimal sedation protocol for future implementation efforts.

## Materials and methods

Study design

This quality improvement protocol implemented the Plan-Do-Study-Act approach to develop the outpatient minimal sedation protocol [26].The initiative took place at University of California, Los Angeles (UCLA) Health (Los Angeles, California), a large, urban university-based health system; higher-level urgent care clinics called Evaluation and Treatment Centers (ETCs) were the targeted outpatient facility. The development of the outpatient minimal sedation protocol required extensive input from a multidisciplinary team of internal medicine physicians, pharmacists, the hospital ambulatory nursing team, and anesthesiologists at UCLA Health. The minimal sedation intervention was implemented at one outpatient clinic within the larger university-based health system. Physicians at the outpatient clinic are trained in both internal medicine and pediatric care (Med-Peds), therefore specializing in care for both children and adults. The clinic has a subset of physicians that specialize in IDD. The clinic’s patient population includes children and adults and a diverse racial, ethnic, and socioeconomic demographic.

Sample

All participants included in the sample were current patients at the outpatient clinic (n=18). Inclusion was based on the physician's clinical assessment of needle phobia, in which the patient reported severe fear of needles and exhibited avoidant behavior or based on medical advice. Patients were recommended for the minimal sedation intervention based on an assessment of routine medical care compliance by their physician. Reasons for referral to intervention included routine lab work, monitoring medication levels, and routine vaccination. The attending physician or registered nurse (RN) obtained consent from the individual or caregiver of the patient. The final sample is based on the number of patients that moved forward with the minimal sedation protocol within the first year of the intervention's assessment (July 2020-July 2021).

The study sample was categorized into two groups: patients with IDD and non-IDD patients. Patients with IDD had a clinical diagnosis of an IDD (e.g., autism, attention deficit hyperactivity disorder (ADHD), cerebral palsy, and intellectual disability) within their medical records. Patients with IDD may or may not have needle phobia (as reported in their medical record or based on a physician's recent clinical assessment). Patients with IDD with and without needle phobia were combined into one group to address the study’s main objective of increasing uptake of needle-related medical procedures among patients with IDD. The non-IDD group (needle phobia only) was considered the comparison group for this study. 

Intervention: minimal sedation (anxiolysis)

Minimal sedation, also known as anxiolysis, is considered a drug-induced state, during which patients respond normally to verbal commands [27]; cognitive function and physical coordination may be impaired, but airway, reflexes, and ventilatory and cardiovascular functions are unaffected. A qualified provider (physician, nurse, or nurse practitioner) administers a fixed dose of 5 mg of intranasal midazolam (benzodiazepine) for older children and adult patients and weight-based dosage of 0.2 mg/kg for smaller children. The maximum dosage to be administered may not exceed 10 mg. After administering the dose, providers wait approximately 10 minutes prior to the patient’s procedure. The medication must be stored in an automated dispensing machine that follows federal and state regulations regarding inventory and disposal of controlled substances. Required equipment within the outpatient facility includes oral airways and suction; oxygen delivery supplies, including appropriately sized ambubag, face masks, nasal cannulae, extension tubing, and connector; a pediatric and adult defibrillator; reversal agents (e.g., flumazenil) as appropriate; and monitoring equipment (continuous pulse oximetry and automated blood pressure cuff). Appendix A includes detailed information on the minimal sedation guidelines used within the study’s clinic.

Measures

To study the feasibility of the minimal sedation intervention, we had two outcomes of interest: (a) administration of sedation was performed, and (b) the medical order(s) (e.g., vaccination and blood draw) were performed. A successful intervention was considered if both sedation and medical orders were performed.

Data collection and analysis

Pilot data on the outpatient minimal sedation protocol were collected at the outpatient clinic from July 2020 to July 2021. De-identified patient demographic data and outcome measures were recorded by the RN administering the protocol. Frequency and descriptive statistics were computed using Stata Statistical Software Release 16.1 (2019; StataCorp LLC, College Station, Texas, United States). The University of California Institutional Review Board considered this study to be exempted from approval (IRB #21-001664).

## Results

Over a 12-month period, the minimal sedation intervention was initiated in the clinic on a total of 18 patients. Patient characteristics and their intended reason for a medical visit are presented in Table 1. Nearly 40% of the patients were female (n=7), and one-third of the patients were younger than 18 years old (33.3%; n=6). Individuals with IDD were 72.2% of patients (n=13). The intended reasons for the visit included to receive routine lab work (83.3%; n=15), receive vaccinations (38.9%; n=7), and/or monitor medication levels (11.1%, n=2). 

**Table 1:** Patient characteristics and intended reason for medical visit IDD: intellectual and developmental disabilities; +: not mutually exclusive

Variable	n (%)
Gender	
Male	11 (61.1%)
Female	7 (38.9%)
Age	
Under 18 years old	6 (33.3%)
18+ years old	12 (66.7%)
IDD diagnosis	13 (72.2%)
Reason for lab visit^+^	
Routine lab work	15 (83.3%)
Therapeutic drug monitoring	2 (11.1%)
Routine vaccination	7 (38.9%)

Table 2 displays the preliminary outcomes from implementing the minimal sedation protocol. Overall, 50% (n=9) of the patients had successful intervention encounters in both administering the sedation and performing the medical orders (Sedation +, Orders +). Nearly one-fourth (22.2%; n=4) of the patients had the sedation successfully administered, but the medical orders were not performed (Sedation +, Orders -). An additional four patients (22.2%) had neither the intended sedation administered nor the intended medical orders performed (Sedation -, Orders -). One patient (5.6%) was able to have orders performed without administering the minimal sedation as intended (Sedation -, Orders +). There were no reported serious adverse reactions to the midazolam.

**Table 2:** Minimal sedation preliminary outcomes (time period=12 months) IDD: intellectual and developmental disabilities

	IDD: n (%)	Non-IDD: n (%)	Combined (IDD and non-IDD): n (%)
Sedation performed and orders performed (Sedation+, Orders+)	5 (38.5%)	4 (22.2%)	9 (50.0%)
Sedation performed and did not perform medical orders (Sedation+, Orders–)	3 (23.1%)	1 (5.6%)	4 (22.2%)
Did not administer sedation and did not perform medical orders (Sedation–, Orders–)	4 (30.8%)	0	4 (22.2%)
Perform medical orders with no sedation (Sedation–, Orders+)	1 (7.7%)	0	1 (5.6%)
Total sample	13 (100%)	5 (100%)	18 (100%)

For patients with IDD, several patients had success in receiving necessary routine care using the minimal sedation intervention (38.4%; n=5); however, four patients (30.8%) did not have sedation administered and did not have medical orders performed (Sedation-, Orders-). Anecdotal reports by the attending RN stated that at the time of the appointment, these patients did not feel adequately relaxed to proceed with the minimal sedation intervention, and therefore the RN did not continue with the intended protocol. In addition, three of the four patients (23.1%) that had sedation administered but did not have the medical orders performed were patients with IDD (Sedation+, Orders-). Similarly, it was reported that after the sedation was administered, these patients experienced sensory overload and did not reach a reasonable level of relaxation to continue with the intervention. Therefore, the attending RN did not continue with the protocol as intended. The Needle Anxiety Program includes clinic guidelines on how providers can prepare for a medical appointment using the minimal sedation protocol.

## Discussion

Overall, this quality improvement pilot study demonstrated that the minimal sedation protocol can improve the performance of routine care for individuals with IDD and/or needle phobia. While many patients will only engage in behavior modification interventions and topical treatments, patients with IDD and/or needle phobia may need the minimal sedation protocol to receive routine care procedures. As demonstrated in the preliminary outcomes, the minimal sedation protocol enables a portion of these patients to receive medical care that was often avoided due to use of needles or injections. In addition, patients who find success in the minimal sedation protocol may continue to use the protocol in managing routine care. Among the current sample, there was one repeat intervention user that had three successful encounters. Within the study’s timeframe, we did not have other participants attempt the intervention more than once. Repeat users/attempts will be considered in future studies of the minimal sedation protocol’s evaluation.

It is important to consider some of the limitations of the minimal sedation protocol prior to implementing it in other healthcare settings. For this initiative, the clinic is not licensed through a hospital system. Therefore, there were fewer procedural approvals than would be needed for facilities licensed through hospital systems; the latter may have difficulty getting the protocol approved due to the interest of more committees wanting to be involved from a safety perspective. Facilities that can implement the minimal sedation protocol are any outpatient settings with RNs on site that can handle a potential overdose (i.e., those that can deliver oxygen and handle a high-level emergency) and can monitor a tight inventory of controlled substances. Therefore, small community offices with nursing assistants may not have the capacity to implement this minimal sedation protocol. Lastly, this study utilized nonprobability sampling and results are not generalizable; additional studies are needed with larger sample sizes to appropriately assess the risks and benefits of the minimal sedation protocol.

While some medical facilities use other sedation strategies to achieve healthcare outcomes in patients with IDD, this minimal sedation protocol has benefits for both providers and patients. Using emergency departments for routine care procedures can be difficult for providers to predict and manage care due to patient volume. Day visits to surgical centers incur operating room charges for patients. Attempting to use this strategy for routine healthcare visits over time can cause a severe financial strain on patients and may lead them to neglect services. Therefore, administering the minimal sedation protocol in outpatient clinics allows more flexibility for the providers and patients and does not strain resources or incur excessive healthcare costs. The minimal sedation protocol allows patients and providers to maintain routine care within the primary care offices.

## Conclusions

The novel minimal sedation protocol for outpatient care assisted in administering needle-related medical care for individuals with IDD and/or needle phobia. This quality report demonstrated the feasibility of the minimal sedation protocol; however, a more rigorous study designed to test the efficacy of the minimal sedation protocol is needed. Future efforts will aim to implement the minimal sedation protocol on a wider scale throughout the university-based healthcare system. We hope this initial report provides insights for other medical facilities aiming to improve healthcare for patients with IDD and needle phobia.

## Appendices

Appendix A. UCLA Health Guideline for Minimal Sedation/Anxiolysis in Ambulatory Care Clinic

Guideline for minimal sedation/anxiolysis in ambulatory care clinic

Purpose

To provide standard for patient care when a single dose of sedative is utilized for routine procedures or tests in an outpatient practice setting for lessening anxiety and discomfort. This policy is used in special practice situations where other options have been explored (i.e., controlling the environment, guarding against sensory overload; distraction; topical anesthetics; consultations with psychologists and child life specialists) to perform the procedure without a sedative and when unsuccessful and the patient would be at risk by not having procedure or test completed.

Scope

This policy shall pertain to patients >/= 2 years of age receiving a single dose of sedative prior to a procedure at [removed] that serves a special patient population with this clinical need.

Definitions

Minimal sedation/anxiolysis: a drug-induced state, during which patients respond normally to verbal commands. Although cognitive function and physical coordination may be impaired, airway, reflexes, and ventilatory and cardiovascular function are unaffected. Defined at UCLA as the administration of one drug, one dose, and one time to be administered by oral or intranasal route.

Qualified provider: physicians, registered nurses (RNs), or nurse practitioners

Requirements: (1) Review of Ambulatory Care Training Module and Competency Validation approved by the Health System Sedation Committee; (2) Current BLS for Health Care Providers

Guidelines

1. Medications must be stored in an automated dispensing machine (i.e., Pyxis machine) following federal and state regulations regarding inventory and disposal of controlled substances.

2. The physician will have primary responsibility to determine the need for minimal sedation/anxiolytics prior to procedure and that there are no contraindications.

3. The physician will order sedation/anxiolytic medication to be administered after exploring other options (distraction, local anesthesia, child life specialist, psychologist, etc.).

4. The physician will determine appropriate medication and dose (see Table 3).

**Table 3:** Dose recommendations for intranasal medication

Drug	Dose	Route
Midazolam	0.2 mg/kg MAX 10 mg	Intranasal

5. The physician or RN will be responsible for the continuous monitoring of the patient during the procedure and clinic stay.

6. The patient will only receive one single dose of medication.

7. The patient will be discharged when clinically stable as defined by discharge criteria and receive discharge instructions based upon medication.

8. A responsible adult must accompany the patient and receive discharge instructions upon medication administered.

9. Pre-treatment with intranasal lidocaine should be considered to reduce pain associated with intranasal midazolam in children.

Procedure

I. Equipment

A. Oral airways and suction

B. Oxygen delivery supplies, including appropriately sized ambubag, face masks, nasal cannulas, extension tubing, and connector

C. Defibrillator: pediatrics and adults

D. Reversal agents as appropriate

E. Monitoring (continuous pulse oximetry, automated blood pressure (BP) cuff)

II. Pre-procedure care

A. Licensed provider to assess and document mental status and vital signs to include the Observer's Assessment of Alertness/Sedation Scale, baseline BP, pulse, oxygen, saturation, respiratory rate (RR), and weight in kilograms.

B. Physician to complete physical examination of the patient and review the patient's medical history to include any known allergies or drug reactions, current medications, and current health issues.

C. Suction any nasal discharge prior to administration of intranasal drug. Intranasal drug should not be used if nasal congestion present. Pre-treatment with topical lidocaine spray should be considered.

III. During procedure care

A. The RN or physician will stay with the patient following the administration dose of the medication and during procedure.

B. Continuous pulse oximetry and pulse monitoring during procedure.

C. Observer's Assessment of Alertness/Sedation Scale, BP, pulse oximetry, RR, and pulse documented every 15 minutes.

IV. Post-procedure care/discharge criteria to home

A. The patient must not be discharged to home until the patient is awake, alert, and oriented to person, place, and time or has returned to their pre-sedation level of consciousness, which includes pre-procedure BP, heart rate (HR), and RR, which must be documented in the patient record.

B. A physician must order discharge after the physician has assessed the patient's readiness for discharge.

C. The patient must be discharged with a responsible adult

D. The responsible adult has received discharge instructions for care at home post-procedure and a number in case of an emergency. This should include necessary observation by an adult post-discharge and may include staying home from school for the remainder of the day.

E. If applicable, the patient is instructed not to drive an automobile or operate heavy machinery until the following morning.
